# Toward a harmonized regulatory framework for 3D-printed pharmaceutical products: the role of critical feedstock materials and process parameters

**DOI:** 10.1007/s13346-025-01966-x

**Published:** 2025-09-16

**Authors:** Riyad F. Alzhrani, Rawan A. Fitaihi, Majed A. Majrashi, Yu Zhang, Mohammed Maniruzzaman

**Affiliations:** 1 Department of Pharmaceutics, College of Pharmacy, King Saud University, Riyadh, 11451 Saudi Arabia; 2 Bioengineering Institute, King Abdulaziz City for Science and Technology (KACST), Riyadh, Saudi Arabia; 3 Pharmaceutical Engineering and 3D Printing (PharmE3D) Lab, Department of Pharmaceutics and Drug Delivery, School of Pharmacy, The University of Mississippi, University, MS 38677 USA

**Keywords:** 3D-printing technology, Personalized medicine, Spritam^®^, Regulatory framework, Point-of-care, Additive manufacturing, Quality control

## Abstract

The commercialization of additive manufacturing (AM) in pharmaceuticals manufacturing has attracted significant attention for its potential to produce customized products. However, the process is slow and hindered by the lack of designated regulatory guidelines tailored to 3D-printed pharmaceutical products (3DPPs). The 3D-printing technology has paved the way for personalized medicine, enabled treatment of rare genetic disorders, and offered many other possibilities for patients. Despite the US Food and Drug Administration (FDA) approval of Spritam^®^, a clear regulatory framework for licensing 3DPPs by the FDA or EMA remains unavailable. The current practice considers all products the same, regardless of their manufacturing method and/or complexity. While this approach has been generally accepted, it frequently fails to evaluate the unique quality attributes of 3DPPs. The lack of a harmonized regulatory framework tailored to the 3DPPs presents a major barrier to the widespread adoption of AM and other innovative technologies. To bridge this gap, this review highlights the most critical parameters related to the feedstock materials and 3D-printing processes, emphasizing their impact on the quality attributes of finished 3DPPs. Numerous scenarios have been proposed to encourage regulatory authorities to establish robust regulatory guidance for the 3D-printing technology at either industrial or point-of-care (PoC) settings. Coordinated efforts between regulatory authorities, industry partners and other stakeholders are necessary to define product specifications and identify appropriate analytical techniques for evaluating finished 3DPPs. By developing a harmonized regulatory framework and establishing quality control measures, the full potential of AM can be realized. This will ultimately ensure that novel 3DPPs and personalized medicines adhere to rigorous regulatory standards of quality, safety and efficacy.

**Graphical abstract:**

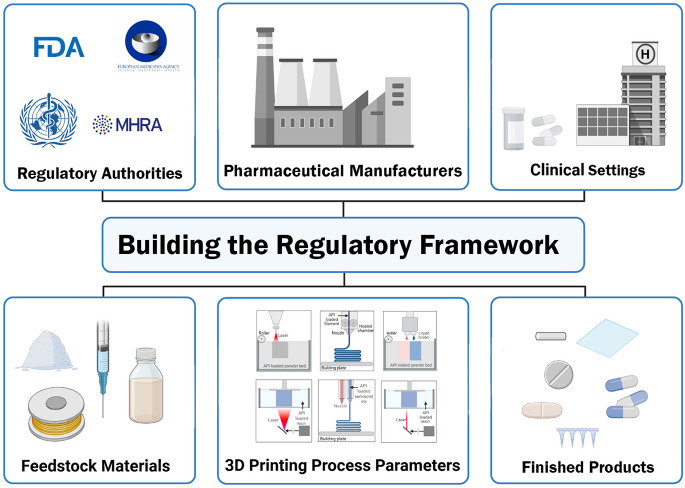

## Introduction

The drug regulatory authority is the paramount governmental body responsible for ensuring the safety and efficacy of pharmaceutical products. This is accomplished by establishing high-quality standards that cover every stage of product development, from conceptualization to post-market surveillance. The pharmaceutical quality is defined as the production of products in compliance with current Good Manufacturing Practices (cGMP) issued by regulatory bodies including FDA, European Medicines Agency (EMA) and International Council on Harmonisation of Technical Requirements for Registration of Pharmaceuticals for Human Use (ICH) [1].

For many decades, the quality of pharmaceutical products was evaluated predominantly through testing the finished product, a method known as a “Quality by Test” (QbT) approach. This method, however, proved impractical due to the high risk of batch failure and the consequent waste of resources [1–3]. To address these challenges, the pharmaceutical industry players, in collaboration with regulatory authorities, have adopted a new concept known as “Quality by Design” (QbD). The QbD approach focuses on identifying potential risks at the early stages of the development process and establishing mitigation strategies to ensure product quality. This approach involves the use of real-time analysis tools to define and monitor desired quality attributes, which in turn can influence the clinical efficacy and safety of the final product [2–4]. By building quality into the product, QbD aims to enhance production efficiency, reduce variability and ensure adherence to regulatory requirements in a science- and risk-based framework [5]. Ultimately, the QbD helps to deliver safe and effective pharmaceutical products to the market.

Innovative technologies, including continuous manufacturing and Three-dimensional (3D) printing technology, have been recognized by regulatory authorities (Table 1). However, regulatory guidance for these technologies often lacks essential details related to their process optimization and product specifications, which contributes to the delay in unlocking the full potential of these technologies [6–12]. It is therefore crucial to establish timely and comprehensive regulatory guidance that identifies critical process steps, defines product specification and sets a list of analytical assessment methods to evaluate the pharmaceutical products manufactured with these technologies.

**Table 1:** List of regulatory documents for innovative technologies in pharmaceuticals research and development

Innovative technology	Regulatory document	Regulatory authority	Release date
Artificial Intelligence (AI)	Reflection paper on the use of Artificial Intelligence (AI) in the medicinal product lifecycle	EMA	2023
	Artificial Intelligence in Drug Manufacturing: Discussion Paper	FDA	2023
Continues manufacturing	Q13 Continuous Manufacturing of Drug Substances and Drug Products	ICH	2023
Advanced manufacturing	Distributed Manufacturing and Point-of-Care Manufacturing of Drugs: Reflection Paper	FDA	2022
Nanomedicine	Drug Products, Including Biological Products, that Contain Nanomaterials	FDA	2022
Gene therapy	Guideline on Good Clinical Practice specific to Advanced Therapy Medicinal Products	EMA	2019
	Considerations for the Design of Early-Phase Clinical Trials of Cellular and Gene Therapy Products	FDA	2015
3D Printing	Technical Considerations for Additive Manufactured Medical Devices	FDA	2017
	Medical Device Regulation (MDR) (EU) 2017/745.	EMA	2017

3D-printing technology has emerged as a transformative approach in pharmaceutical manufacturing, offering unlimited flexibility in drug product design, dosage customization and personalized medicine. This technology holds significant promise in various therapeutic areas including pediatrics, geriatrics and rare genetic disorders [13–15]. Despite its promise, the integration of 3D printing into pharmaceutical product manufacturing presents regulatory challenges due to the novel nature of the technology and the wide range of variability expected in personalized medicine. A major milestone in this field was marked when the FDA approved the first 3D-printed pharmaceutical product, Spritam^®^ [16]. Surprisingly, there is still no clear regulatory framework for licensing 3DPPs [7, 17]. It is imperative for regulatory authorities to develop a harmonized regulatory framework to enable the extensive adoption of 3D-printing technology in pharmaceuticals research, development and translation.

## Current regulatory framework of 3D-printed pharmaceutical products

Spritam^®^, an epilepsy therapy, represents the first 3D-printed pharmaceutical approved by the FDA under the Emerging Technology Program (ETP) in 2015 [18, 19]. This regulatory pathway is designed to facilitate the approval of novel technologies and complex products, such as pharmaceutical products fabricated using 3D-printing technology (Table 2). Unfortunately, uncertainty remains regarding the regulatory requirements for quality testing and approval of 3D-printed pharmaceutical products [17, 20]. The current standards for the FDA consider all pharmaceutical products under similar regulatory standards outlined in the 21 CFR Part 210 and 211. However, this traditional practice may not adequately address the unique quality attributes associated with 3D-printed pharmaceutical products. Studies have highlighted that some critical quality attributes of 3D-printed products cannot be effectively assessed using the current ICH Q6 guidelines. While ICH Q6 guidelines effectively address traditional quality attributes such as content uniformity, dissolution and degradation products, they do not fully cover CQAs unique to 3D-printed finished products. These include parameters such as structural fidelity, layers adhesion strength, spatial distribution of APIs and layers resolution, which are essential for ensuring 3D-printed product quality [21–23]. Due to high demand for 3D-printing technology in fabricating patient-specific devices, the FDA issued a guidance document titled “Technical Considerations for Additive Manufactured Medical Devices,” which highlighted technical considerations related to additive manufacturing and emphasized on meeting the regulatory requirements for medical device authorization [26, 27]. In parallel, point-of-care (PoC) drug manufacturing is being actively encouraged, and recent feedback from stakeholders is helping to facilitate the release of final guidance [24]. While these efforts represent a starting point for the FDA, there is still an immediate need for tailored guidance for 3D-printed pharmaceutical products.

**Table 2:** Number of applications received by the emerging technology program (ETP)

Technology type	Number of applications	Examples
Continuous Manufacturing	46	• Continuous manufacturing of drug product. • Ultra-long-acting oral formulation. • Model-based control strategy for continuous manufacturing
3D printing technology	-	• New drug products (Spritam^®^ 2015)
New Aseptic	11	• Novel container and closure systems for injectable products • Closed aseptic filling system
Novel Analytical	12	• Multi-attribute method

The EMA has not established a regulatory framework for 3D-printed pharmaceuticals, nor has it authorized any 3D-printed product. However, the EMA’s Council Directive (2017/745) concerning medical devices has been a long-standing framework for developing medical devices for human use [25]. This guidance offers insights into the regulations of medical devices and utilizes a risk-based approach similar to the FDA. In addition, the Innovative Task Force (ITF) is a designated group of experts from the EMA and industry with the goal of identifying solutions for challenges associated with novel technologies, including 3D-printing technology [26]. Similar to the FDA, there is preliminary regulatory guidance covering some aspects of 3D-printing technology but there is a lack of comprehensive regulatory guidance for 3D-printed pharmaceutical products.

To establish such robust regulatory oversight, it is very important to apply the QbD concept for the development of 3D-printed pharmaceutical products. The QbD is a well-established regulatory practice designed to improve the efficiency of pharmaceutical development by closely monitoring both input and output parameters [2, 3]. The QbD methodology centers around four essential elements: target product profile (TPP), critical process parameters (CPPs), critical material attributes (CMAs), and critical quality attributes (CQAs). An in-depth understanding of a product development process is utilized frequently to identify CMAs and CPPs, which in turn define the CQAs of the finished pharmaceutical product. Continuous control and optimization of these properties ensure that the production of pharmaceutical products consistently achieves their prespecified TPP [2, 3, 5].

In the context of 3D-printing technology, there are two critical key players that can impact the quality of 3DPPs: feedstock material and 3D-printing process. The physicochemical properties of the printable inks, often composed of active pharmaceutical ingredients (APIs) and pharmaceutical-grades excipients, vary based on the 3D-printing technology employed. These properties represent key CMAs that influence the finished product’s quality attributes [27]. Furthermore, the 3D-printing process involves multiple stages and requires careful optimization to ensure the fabrication of high-quality pharmaceutical products, which can be time-consuming [28, 29]. Post-processing of 3DPPs can introduce additional challenges which may affect the finished product quality characteristics [29].

**Fig. 1:**
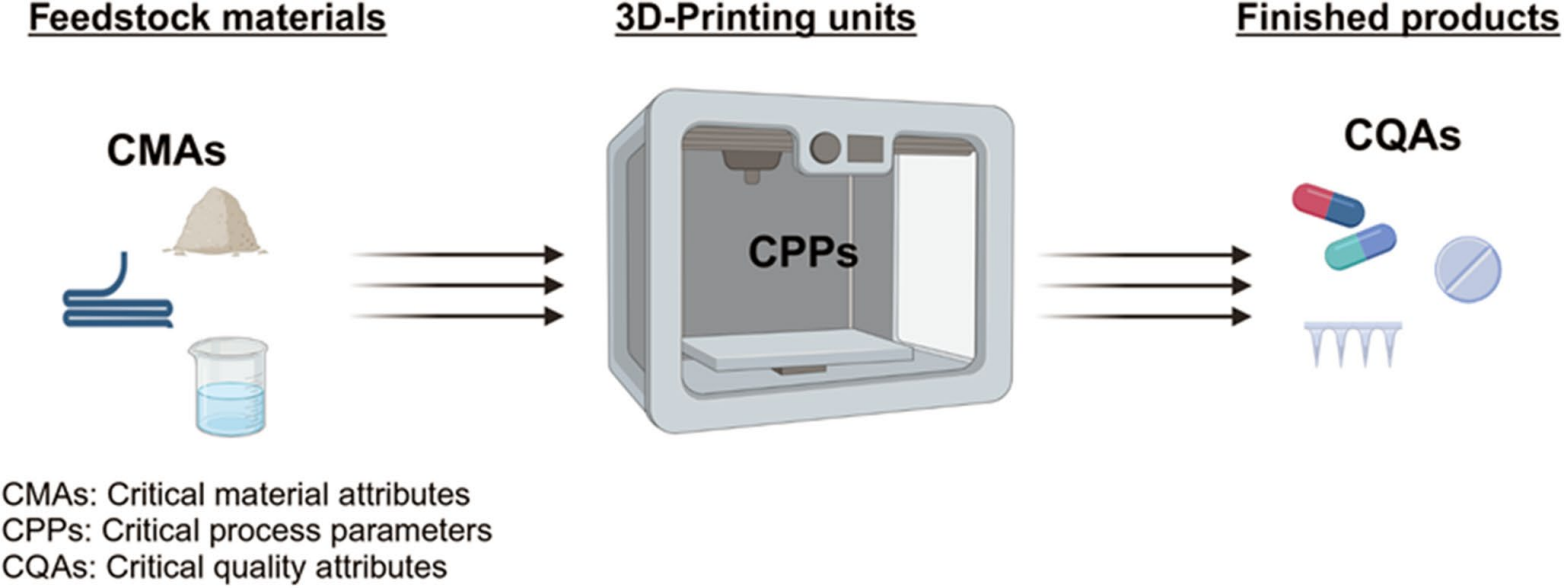
The relationship between feedstock materials and the 3D-printing process parameters is a major key in defining the critical quality attributes of finished products

Due to the lack of comprehensive guidance on the CPPs and CMAs that directly influence CQAs of the finished product (Fig. 1), this work aims to bridge this gap by compiling a list of the CQAs of pharmaceutical products fabricated with different 3D-printing technologies. This initiative will provide a foundation for regulatory authorities to develop regulatory framework and ultimately ensure that the 3DPPs meet regulatory expectations.

## Introduction to 3D-printing technology

3D-printing is an innovative AM technology that relies on constructing objects through a layer-by-layer deposition mechanism. From rapid prototyping to large-scale manufacturing, this technology has been very effective in providing many innovative solutions in various industries, including automotive, aerospace, and biomedical sectors [30–32]. In pharmaceutical manufacturing, the 3D-printing technology enabled the fabrication of complex dosage forms with different geometries, rough surfaces, and hollow structures, which are typically very challenging to produce with traditional manufacturing processes. Multilayer tablets, caplet in caplet and floating capsules are all novel examples of 3D-printed pharmaceutical products [30–36].

Beyond these advancements, PoC manufacturing is another direction of the 3D-printing technology due to its capability to fabricate pharmaceutical products at hospitals or pharmacies. These products can be designated to treat a specific disease condition, such as orphan diseases, in which a small-scale batch is sufficient [37, 38]. Furthermore, the 3D-printing technology may pave the way for personalized medicine by enabling pharmaceutical product customization in terms of drug type, dose, and release rate. This is very beneficial to patients to get faster access to therapy, better efficacy and ultimately lower side effects.

The 3D-printing process encompasses various fabrication techniques, materials, and layering procedures [39]. This process becomes even more complicated when APIs with different physicochemical properties are loaded into the material before printing. The 3D-printing workflow can broadly be considered to involve three main stages. The first stage includes both the preparation of printable feedstock material such as API-excipient mixtures and creating a specific design containing the desired size, shape and internal structure of the pharmaceutical product. Then, the design file is converted into a Standard Tessellation Language (STL) file, which is then transldated inot G-code, the readable fromat by 3D printers. Finally, the final object is constructed. Post-process treatment, such as curing or drying, may be necessary before the collection of the finished 3DPPs [40].

### Feedstock material and 3D-printing process control

The development of the right feedstock material is an essential preliminary step to enable the transformation of raw materials to 3D finished products suitable for patient use. The inclusion of specific excipients, e.g., polymers and lubricants, with the API is often required for the preparation of the feedstock material in the physical form of a powder, filament, or solution, depending on the type of 3D-printing technology used [27]. The successful development of this step is also important for the production of 3D-printed pharmaceutical products with a specific drug content release rate and confer protection for the API during and after the 3D-printing process [41, 42]. For instance, it is unlikely to prepare a filament with thermolabile drugs without expecting impurities and potency loss from the heated printhead used in the extrusion-based 3D-printing technology [43]. Additionally, the balance between the raw material formula and printability parameters should be achieved for the ideal situation of 3DPPs. The filament’s glass transition temperature, mechanical strength and elasticity are examples of printability parameters for feedstock material used in extrusion-based 3D printing technology [44, 45]. When fabricating a pharmaceutical product using different feedstock materials, additional requirements regarding the compatibility of building materials should be thoroughly monitored. These aspects define the CMAs of the feedstock material, which are very critical for maintaining the reproducibility, accuracy and reliability of the 3D-printing process.

In addition to CMAs, various general CPPs related to the 3D-printing process, including printing speed, printing pattern, infill density and layer height, play a major role in the finished product’s quality attributes and characteristics. Failure to optimize these parameters can result in defects, variable layer thickness and ultimately failure to make a pharmaceutical product that satisfies quality standards [46, 47]. An illustrative example of this is the poor resolution commonly seen in finished products when the 3D-printing speed is not optimized. Nevertheless, each 3D-printing technology has its own CPPs that have to be met, including controlling the binder viscosity and temperature of the nozzle for the binder jetting and extrusion-based 3D printers, respectively. Unlike CMAs, CPPs can be considered a critical control point of the 3D-printing process that, when not well-controlled and continuously assessed, will lead to process failure and significant resource loss, particularly in large-scale manufacturing settings [48].

### 3D printer specifications

One of the key regulatory considerations for the use of 3D-printing technology in pharmaceutical manufacturing is the requirements regarding the 3D printer itself. 3D printers used in the production of pharmaceutical products must meet stringent standards for cleanliness, accuracy, and reliability to ensure the consistent and reproducible production of drug products [49, 50]. These 3D printers must also be validated to ensure that they are capable of producing the desired pharmaceutical products with the intended quality attributes [51]. Printer validation involves installation qualification (IQ), operational qualification (OQ), and performance qualification (PQ) to ensure the printer operates correctly and consistently to produce high-quality pharmaceutical products.

The hardware of the 3D printer must be designed and constructed to prevent contamination, maintain dimensional accuracy and operate according to cGMP standards. This includes the use of materials that are compatible with the drug product and the manufacturing environment, along with optional features such as sealed enclosures, air filtration systems, controlled temperature and humidity. Furthermore, implementing physical barriers, using allocated printer component for different APIs and enforcing critical cleaning protocol ultimately minimize the cross contamination [49, 52].

The manufacturing process for 3DPPs must be carefully controlled to ensure the production of safe and effective pharmaceutical products. This includes the utilization of an appropriate bulk material mixer to prepare the feedstock material, the implementation of in-process monitoring tools, including the use of real-time sensors to monitor CPPs, as well as establishing testing criteria for the finished pharmaceutical products [45, 53]. With the integration of real-time process analytical technology (PAT), CQAs such as weight variation and dissolution testing, can be obtained instantaneously and provide an insight on the performance of the manufacturing process [54, 55].

### Type of 3D-printing technology

3D-printing technologies are generally classified into seven on the basis of the guidelines issued by the ISO/ASTM [13]. However, with the rapid technological advancement, new 3D-printing technologies have surfaced, such as digital light processing (DLP) and direct powder extrusion (DPE), but their printing mechanism is relevant to the following 3D-printing technologies: direct energy deposition, vat polymerization, fused deposition modelling, selective laser sintering, binder jetting, material jetting, and sheet lamination [13, 39]. In this review, we classified the 3D-printing technologies into three categories: powder-based, extrusion-based, and vat photopolymerization-based 3D printing technology. This classification aims to improve clarity by grouping technologies which exhibit a similar underlying printing mechanism and/or use similar feedstock material for the 3DPPs.

#### Powder-based 3D-printing technology

**Fig. 2:**
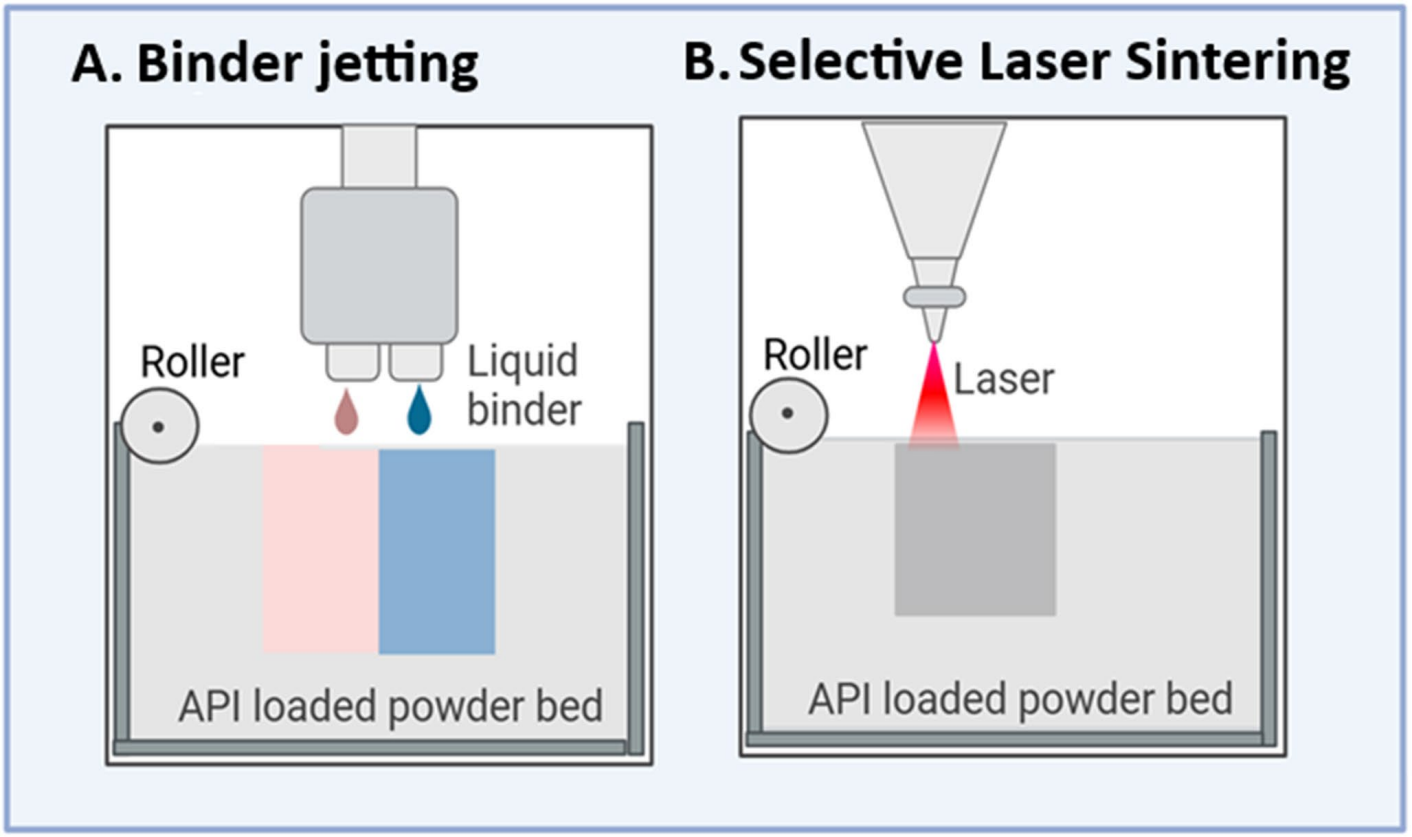
Simplified diagram for two types of powder-based 3D printing technology: Binder jetting (**A**) and Selective. Laser Sintering (**B**)

Powder-based 3D printing is one of the earliest technologies adopted for pharmaceutical product manufacturing. There are two classes of this technology: binder jetting and selective laser sintering (SLS) (Fig. 2). Several novel manufacturing techniques and drug products have been developed with powder-based 3D printing because of its versatility, compatibility and scalability [56, 57]. A notable example is ZipDose, which is a binder jetting 3D-printing technology pioneered by Aprecia Pharmaceuticals. This technology is employed today in the commercial production of Spritam^®^, orally disintegrating tablets (ODTs) that rapidly disintegrate in the mouth of patients with difficulty swallowing [16]. The primary feedstock material consists predominantly of powders and excipients, closely resembling the raw materials used in the conventional manufacturing of oral tablets [58]. Moreover, the incorporation of APIs into the powder is a straightforward process, and there is a lack of a significant modification step for the feedstock material, such as converting the powder to a filament as prerequisite step for the extrusion-based 3D printing technology [59, 60].

For successful printing of a pharmaceutical product, careful attention should be directed to the CMAs of the powders when mixed with APIs. The powder should demonstrate good flowability, compatibility with APIs and stability during as well as after 3D-printing [61]. The binder jetting and SLS share similar process parameters that are appropriately monitored to ensure reproducibility, precise dosage and produce a pharmaceutical product with defined CQAs. Unlike other 3D printing technologies, the powder-based 3D printing constructs objects without the need for a building plate. It also allows the reuse of unprocessed powder, thereby minimizing material loss and reducing production costs [57, 60].

##### Binder jetting

Binder jetting (BJ) is a process of selectively depositing liquid droplets onto a bed containing powder, which is subsequently solidified in layers, forming a 3D-object. This technology has been predominantly used for developing various pharmaceutical products with unique properties, including fast disintegrating oral tablets and multilayered tablets customized for patients’ needs [59, 60]. This technique, patented in 1993 by Emanuel Sachs, has also received attention in fabricating drug-eluting devices [60]. The printing process involves three critical stages: [1] droplet deposition [2], powder interaction and [3] solution drying (Fig. 2A). Optimizing CPPs, including nozzle diameter, roller speed and droplet-specific parameters, is of great importance to obtain consistent droplet deposition (Table 3). The binder jetting nozzle diameter directly impacts droplet volume, and proper control is necessary to avoid clogging and/or defects in the pharmaceutical products such as splashing or coffee staining [62, 63].

**Table 3:** Quality risks associated with poorly optimized critical process parameters (CPPs) and critical material attributes (CMAs) in powder-based 3D printing technology

Binder jetting CPPs	Quality risks	CMAs	Quality risks
Nozzle diameter	Clogging of printing heads, incomplete printing	Binder rheological properties (e.g., surface tension, viscosity)	Inconsistent shape, droplet formation failure
Roller speed	Variable layers thickness, dose variation, poor layers adhesion, collapse	Powder characteristics (e.g., morphology, particles size)	Low printing resolution, weak structural integrity
Droplet-related properties (e.g., volume, jetting rate, spacing)	Poor layers adhesion, high moisture content, weak structural integrity	API (e.g., binder solution stability, solubility, powder compatibility)	Drug degradation, impurities, crystallization, irregular release rate

Selective laser sintering CPPs		CMAs	
Laser-related properties (e.g., intensity, scan speed, scan spacing)	Variable mechanical properties, low printing resolution, longer processing time	Powder characteristics (e.g., particles size, flowability, packing density)	Variable layers thickness, powder shrinkage, porosity, irregular release rate
		API (e.g., laser stability, thermal resistance, powder compatibility)	Drug degradation, impurities, polymorphism, complexation with polymer

Poor layer adhesion: Inadequate adhesion between the successive layers of the 3D-printed product

Variable layer thickness: Inconsistency in the size and composition of layers of the 3D-printed product

Low printing resolution: Lack of fine details in surface and properties of 3D-printed product

Dose variation: Inconsistency between the drug dose between the 3D-printed products

Irregular release rate: Alteration in the drug release rate

CMAs also have a major implication on the resolution and layer thickness of pharmaceutical products, particularly when a precise dosage of an API is needed. To fabricate a pharmaceutical product, there are two options to incorporate APIs: either in the powder mixture or in the binder solution, depending on their stability and compatibility with the printing process. Attributes such as powder flowability, particle size, binder viscosity and surface tension should be closely evaluated to prevent many potential defects in the finished product [61, 64, 65]. Studies have reported that the surface tension of the binding droplet solution prepared from organic solvent should be around 35–40 mJ N^− 1^ [57]. The main advantage of binder jetting is the lack of high-temperature or laser source during the fabrication process, suggesting a feasible 3D printing technology for printing thermolabile APIs [60]. Nevertheless, pharmaceutical products prepared with the binder jetting are typically not readily available for use. They require a post-printing process of solvent evaporation at controlled conditions and removal of unbound powder using methods such as vacuum or air-blowing. These steps are essential for ensuring product uniformity, long-term stability and batch reproducibility [66, 67].

##### Selective laser sintering

SLS is the 2nd class of the powder-based 3D printing technology. Instead of using a binder solution, it utilizes a laser beam that causes the fusion of powder particles. During the laser-powder interaction, the temperature increases, causing the powder particles to sinter together and form a porous layer. After each layer is constructed, a new unprocessed powder is recoated over the previous layer and the same procedure is repeated again (Fig. 2B). Due to its versatility, the SLS-based 3D printing technology has been employed to fabricate oral pharmaceuticals with various product shapes and drug release rates [56, 68]. In addition, many tissue scaffolds and medical devices have been fabricated with the use of SLS [69].

There are many CPPs related primarily to the radiation source, such as laser speed and laser scanning pattern, which can modulate the release rate of API in the finished product (Table 3). In a recent study, orally disintegrating printlets prepared from (HPMC E5) and vinylpyrrolidone-vinyl acetate copolymer (Kollidon^®^ VA 64)) showed a proportional dependence between the laser scan speed and the release rate of the drug. The dissolution of paracetamol changed dramatically, from 60 to 10 min, when the laser speed increased from 100 to 300 mm/s, respectively [70]. This can be attributed to the highly porous surface structure of the printlets fabricated at higher laser speed. Under these conditions, other quality attributes of the orally printed tablets, such as hardness and friability, are expected also to alter.

Similar to the binder jetting 3D printers, the properties of the powder, e.g., particle size and morphology, remain among the most CMAs [71, 72]. For instance, particle sizes between 45 and 90 μm are recommended for optimal packing density and high printing resolution. Whereas smaller particle sizes offer better resolution, they are often suffering from poor powder flowability, and thus it makes them more challenging to handle [72]. Special attention should be directed to the physicochemical properties of the powder when it is formulated with APIs, as the latter may interfere with the printability of the thermoplastic powder. The exposure of APIs to thermal decomposition is considered a limiting factor for the translation of the SLS in the pharmaceutical area [68].

#### Extrusion-based 3D-printing technology

**Fig. 3:**
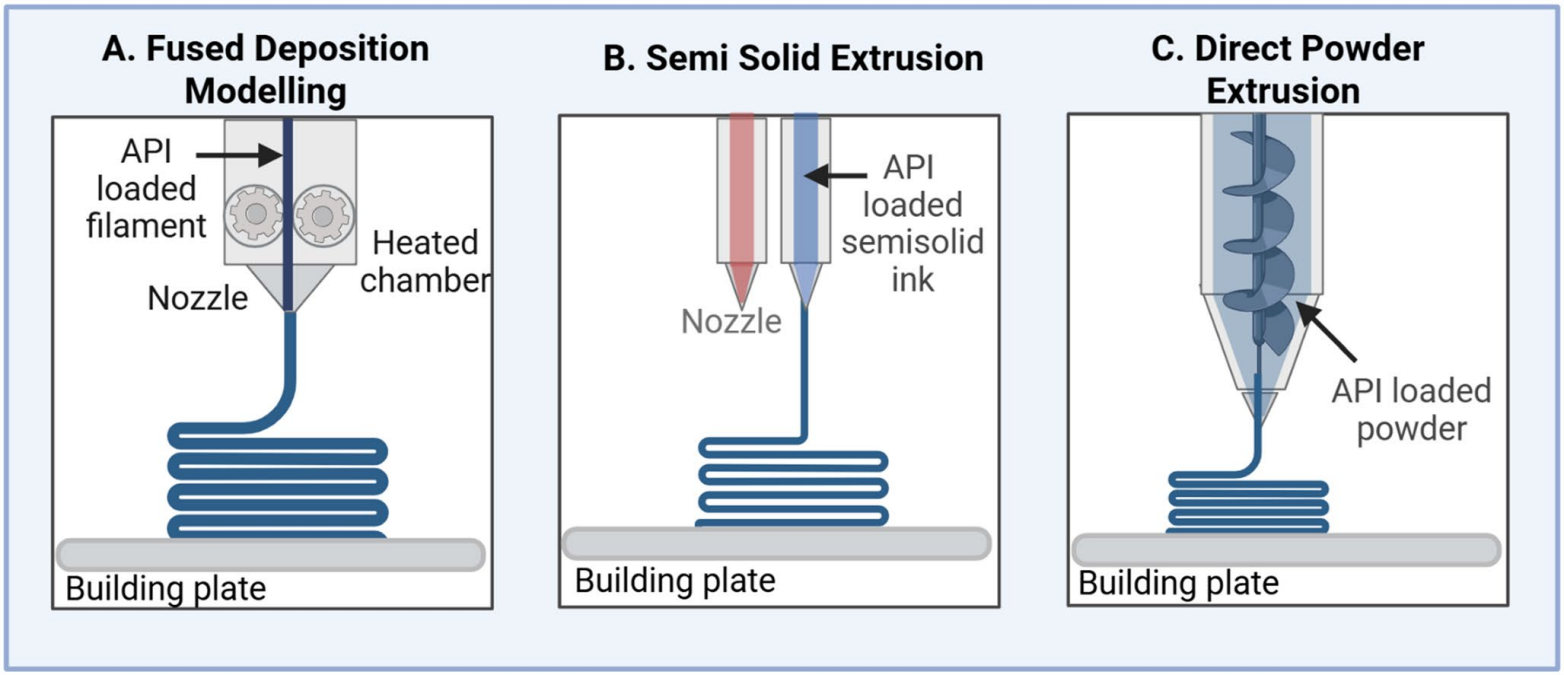
Simplified diagram for three types of extrusion-based 3D printing technology: Fused Deposition Modeling (**A**), Semisolid Extrusion (**B**) and Direct Powder Extrusion (**C**)

The printing process of extrusion-based 3D printing involves depositing API-loaded material onto a building plate using a pressure-based system. However, the raw materials require significant pre-processing before becoming suitable for extrusion-based 3D printing. Screening for the appropriate excipients is very important to define the quality attributes of 3D-printed pharmaceutical products, such as dissolution rate and stability [73]. There are three main classes of extrusion-based 3D printing technology: fused deposition modeling (FDM), semi-solid extrusion (SSE), and direct powder extrusion (DPE) (Fig. 3). While these methods work by the same fabrication mechanism, they differ in the types of feedstock materials utilized, which are usually a thermoplastic polymer, gel, or powder, respectively [74–76].

Each of these techniques is characterized by unique CMAs, but they share many similarities in their CPPs, including nozzle temperature and printing speed (Table 4). As above-mentioned, the 3D-printing process relies on layering material onto the printing bed, where it solidifies to form a pharmaceutical product with specific CQAs [13, 14]. Failure to adequately control the printing bed temperature or the material solidification process may potentially result in quality defects after 3D printing.

**Table 4:** Quality risks associated with poorly optimized critical process parameters (CPPs) and critical material attributes (CMAs) in extrusion-based 3D printing technology

Fused deposition modeling CPPs	Quality risks	CMAs	Quality risks
Nozzle temperature	Poor layers adhesion, clogging of printing heads, incomplete printing	Filament properties (e.g., mechanical, thermal)	Filament degradation, extrusion failure, solidification issues
Printing speed	Poor layer adhesion, low printing resolution	Filament diameter	Dose variation, variable weights
Bed temperature	Warping, inconsistent shape, detachment	API (e.g., filament stability, excipients compatibility)	Drug degradation, impurities, irregular release rate.

Semisolid extrusion CPPs		CMAs	
Nozzle diameter	Low printing resolution, clogging of nozzle	Semisolid material properties (e.g., extrudability, rheology, viscosity)	Variable layer thickness, low printing resolution, shrinkage, collapse
Printing pressure	Poor layer adhesion, low printing resolution	Polymer content	Variable weight, weak structural integrity, irregular release rate
		API (e.g., semisolid material solubility, stability, excipients compatibility)	Drug degradation, impurities, irregular release rate

Direct powder extrusion CPPs		CMAs	
Screw rotation speed	Dose variation, inconsistent shape, variable weight	Powder characteristics (e.g., particle size, viscosity, flowability, density)	Poor layer adhesion, porosity, extrusion resistance, irregular release rate, solidification issues.

Poor Layer adhesion: Inadequate adhesion between the successive layers of the 3D-printed product

Variable Layer thickness: Inconsistency in the size and composition of layers of the 3D-printed product

Low resolution: Lack of fine details in surface and properties of 3D-printed product

Dose Variation: Inconsistency between the drug dose between the 3D-printed products

Irregular release rate: Alteration in the drug release rate

Warping: Curving at the edge of the 3D-printed product

Solidification issues: Inability of 3D-printed product to become solid after post-printing treatment

##### Fused deposition modeling

The FDM enables the building of a pharmaceutical product by melting and layering a thermoplastic filament over a printing bed (Fig. 3A). Due to its cost-effectiveness, FDM has received a considerable attention for the development of drug-eluting devices and scaffolds [44, 74]. The translation of this technology has also been directed to the fabrication of various oral-based pharmaceutical dosage forms with immediate, pulsatile and controlled drug release [77]. Notable examples of successfully printed pharmaceuticals with FDM include gastro-retentive floating tablets, polypills and microneedles [78–82]. To print a pharmaceutical product via FDM, an in-house formulation of API with a thermoplastic polymer is extruded into a filament using Hot Melt Extrusion (HME) techniques, frequently at the diameter range of 1.75 to 3.00 mm [78, 83, 84]. In fact, coupling the HME technique with FDM has been widely adopted to enhance the dissolution of poorly water-soluble APIs following its incoroporation into the polymeric matrix of the feedstock material. Specific excipients, such as lubricants and plasticizers, might be necessary to facilitate the extrusion of filaments during both the HME and 3D-printing processes [85, 86]. The CMAs of the feedstock material, mainly the filament mechanical and rheological characteristics, must be continuously assessed to ensure high-quality 3DPPs (Table 4). Given the presence of the API within the filament, various solid-state analyses are frequently performed to evaluate changes in the quality attributes of products constructed via FDM [44].

Various studies have confirmed that CPPs of the FDM printing process, including nozzle temperature, printing speed and bed temperature, have a major influence on the quality attributes of finished pharmaceutical products [87, 88]. The printing speed has been documented to have a direct impact on the weight uniformity and porosity of the 3D-printed product [89]. Moreover, nozzle printing temperature, which is defined by the thermal characteristics of the filament loaded with API, is a high-risk step for the successful 3D-printing of a pharmaceutical product. This step, if not carefully controlled, can cause thermal degradation of the API along with the risk of compromising the safety of the product [44, 90]. This issue was observed recently with the 3D-printing of tablets containing enalapril maleate extruded into a filament of Soluplus^®^ and Eudragit^®^ E PO. Whereas the HME process has a minimal degradation effect on the enalapril maleate, the 3D-printing process, in which the nozzle temperature was set to 180 °C, led to the development of a high amount (∼ 50%) of the diketopiperazine (DKP) impurity, particularly with Soluplus^®^. Additionally, it was found that nozzle diameter and printing speed had a negligible effect on the degradation of enalapril maleate [43, 44]. Despite the wide feasibility of FDM in the pharmaceutical’s development and biomedical applications, this technology has many challenges related to identifying biodegradable and safe feedstock material for human use.

##### Semi-solid extrusion

Semi-solid extrusion (SSE) works in a similar fashion to FDM but utilizes a different feedstock material. This material, typically a highly viscous semi-solid, is often prepared by mixing polymer and excipient, either with or without the use of a solvent, depending on formulation requirements. It is then loaded into a syringe and 3D-printed onto a building plate at low temperatures (Fig. 3B). There are many other terms referred by authors to the SSE including but not limited to material extrusion, pressure-assisted microsyringe (PAM) and direct ink writing [91–93]. The SSE is a widely recognized 3D printing technology with several applications in biomedical engineering [75, 94]. Due to the lack of high temperature requirements, this method enables the 3D printing of thermosensitive macromolecules and APIs in a product [95]. For successful printing using SSE, APIs are typically dissolved or dispersed within a gel or paste matrix before printing. Orodispersible films, personalized chewable tablets and multicompartmental tablets are examples of pharmaceutical products fabricated via the SSE [96–99]. Several key requirements must be met for the feedstock material to be printable. Among these, viscosity and rheological properties represent the most important CMAs that significantly influence both the 3D printing condition and final product’s quality attributes (Table 4). The ideal rheological property of the semi-solid material should exhibit shear-thinning behavior, where the material behaves like fluid under printing pressures and quickly recovers its elasticity after the printing process [100–102]. This ensures the material maintains its structure following printing and prevents deformation during solidifications.

Optimization of the feedstock material is necessary to obtain specific attributes in the final products [95]. A recent study by Tagami et al. concluded that the polymer content in the feedstock material modulated several quality attributes of 3D-printed tablets, such as dissolution rate, hardness and weight. Increasing the amount of hydroxypropyl methylcellulose (HPMC) retarded the release of naftopidil and reduced tablet hardness [96]. While it is possible to examine some quality attributes of 3D-printed pharmaceutical products using standardized pharmacopoeia tests, several unique attributes, including friability and mechanical strength, may not be appropriately tested [21, 31]. The impact of the CPPs on the 3D-printed pharmaceutical quality attributes is closely similar to the FDM. Nozzle diameter, as well as controlling the pressure, has been predominantly documented to influence the printing resolution of the final product [101, 103]. Unlike the FDM, 3D-printed pharmaceuticals with SSE sometimes require post-printing drying or curing under UV light, posing a risk of product shrinking or deformation while solidifying [94].

##### Direct powder extrusion

It is a novel 3D-printing technology that has received considerable attention for the development of personalized medicine [76]. In this technology, the feedstock is a powder mixture of excipient and API that are directly printed in a single step, eliminating the necessary step of extruding the API into a filament for the FDM. The direct powder extrusion (DPE) system is equipped with a single extruder connected to a printhead nozzle. During the 3D printing process, the powder containing API is melted and extruded through the printhead nozzle, forming the desired pharmaceutical product (Fig. 3C). In comparison with the FDM, the DPE is an appropriate method for the small-scale production of unique pharmaceutical products in the clinic [104, 105]. Relevant examples include abuse-deterrent tablets, immediate-release tablets and personalized minitablets [106, 107]. Additionally, the DPE enables the 3D printing of costly APIs and excipients for specific populations, such as rare diseases [76].

The DPE can be considered a single-unit HEM-FDM 3D printing technology, and therefore, they share a lot of similarities in terms of CMAs and CPPs (Table 4). However, powder flowability appears to be a major CMA for the feedstock material used in the DPE [108]. The high electrostatic interaction between the powder and screw metal surface may hinder the movement of powder toward the printhead and ultimately lead to printing failure [109, 110]. Dry granulation and/or using pellets may be employed to overcome this limitation [104, 108, 111]. Regarding to the CPPs, it is very reasonable to assume that the screw rotation speed is a critical elements of the printing process and it should be monitored to ensure the homogenous melting of the powder before the nozzle extrusion [112]. Another CPP in DPE is the extrusion multiplier, which determines the actual volume of material extruded. It directly influences the quantity of material extruded, hence impacting printing resolution [76].

This technology is relatively new, with only a few 3D-printed pharmaceutical products. Nevertheless, the DPE hold many potentials to become a practical tool for producing customized medicine at the clinic as well as in a manufacturing site [104]. Melt Extrusion Deposition (MED™) is an example of versatile DPE-based 3D printing technology that is currently leveraged by Triastek Inc. with the annual capacity of more than 70 million units [113, 114].

#### Vat photopolymerization-based 3D printing technology

Vat photopolymerization is a novel fabrication technique with diverse medical applications [14, 115]. The fabrication of this process is based on inducing crosslinking in a polymeric resin containing a mixture of monomers, or photopolymers, with the presence of a photoinitiator. This process is triggered by light radiation that cures the photopolymers, converting the material from a viscous liquid to a solid structure [115, 116]. The vat photopolymerization is one of the most explored 3D printing technologies in tissue engineering and medical devices for fabricating products with a high degree of complexity. Different vat photopolymerization-based 3D printing techniques have been developed, including but not limited to stereolithography (SLA), digital light processing (DLP), liquid crystal display (LCD) and continuous light interface production (CLIP) [39, 115, 117]. However, tremendous efforts have been directed lately to extend the application of the vat photopolymerization based 3D printing technology in pharmaceuticals translation due to mainly its high resolution, accuracy and reproducibility. These elements are the key to the manufacturing of pharmaceutical products with high quality standards. In vat photopolymerization 3D-printing technology, several CMAs and CPPs contribute to the CQAs of the finished product (Table 5). Significant attention should be paid to these parameters to deliver a pharmaceutical product with unique quality attributes such as specific drug release rate and/or drug combination.

**Table 5:** Quality risks associated with poorly optimized critical process parameters (CPPs) and critical material attributes (CMAs) in vat photopolymerization-based 3D printing technology

Stereolithography CPPs	Quality risks	CMAs	Quality risks
Laser-related properties (e.g., intensity, scan speed, spacing)	Low crosslinking density, variable layer thickness	Polymer/monomer solution properties (e.g., rheology, molecular weight, functional groups)	Low printing resolution, low crosslinking density, poor layer adhesion, dose variation
Exposure time	Inconsistent shape, weak structural integrity	Photoiniator content	Low printing resolution, poor layer adhesion, low crosslinking density
		API (e.g., resin stability, solubility, excipients compatibility)	Drug degradation, impurities, irregular release rate

Digital light processing CPPs		CMAs	
Laser intensity	Low printing resolution, weak structural integrity	Polymer content	Printing failure, weak structural integrity
		API (e.g., stability and solubility)	Low loading efficiency, poor crosslinking

Poor Layer adhesion: Inadequate adhesion between the successive layers of the 3D-printed product

Variable Layer thickness: Inconsistency in the size and composition of layers of the 3D-printed product

Low printing resolution: Lack of fine details in surface and properties of 3D-printed product

Irregular release rate: Alteration in the drug release rate

There are several hurdles that need to be overcome to increase the translation vat photopolymerization in pharmaceutical manufacturing. Safety of photopolymers remains a major limiting factor, and extensive studies should be performed to ensure the products, after photopolymerization, are free from toxic free radicals, by-products and unreacted polymers [118].

##### Stereolithography

**Fig. 4:**
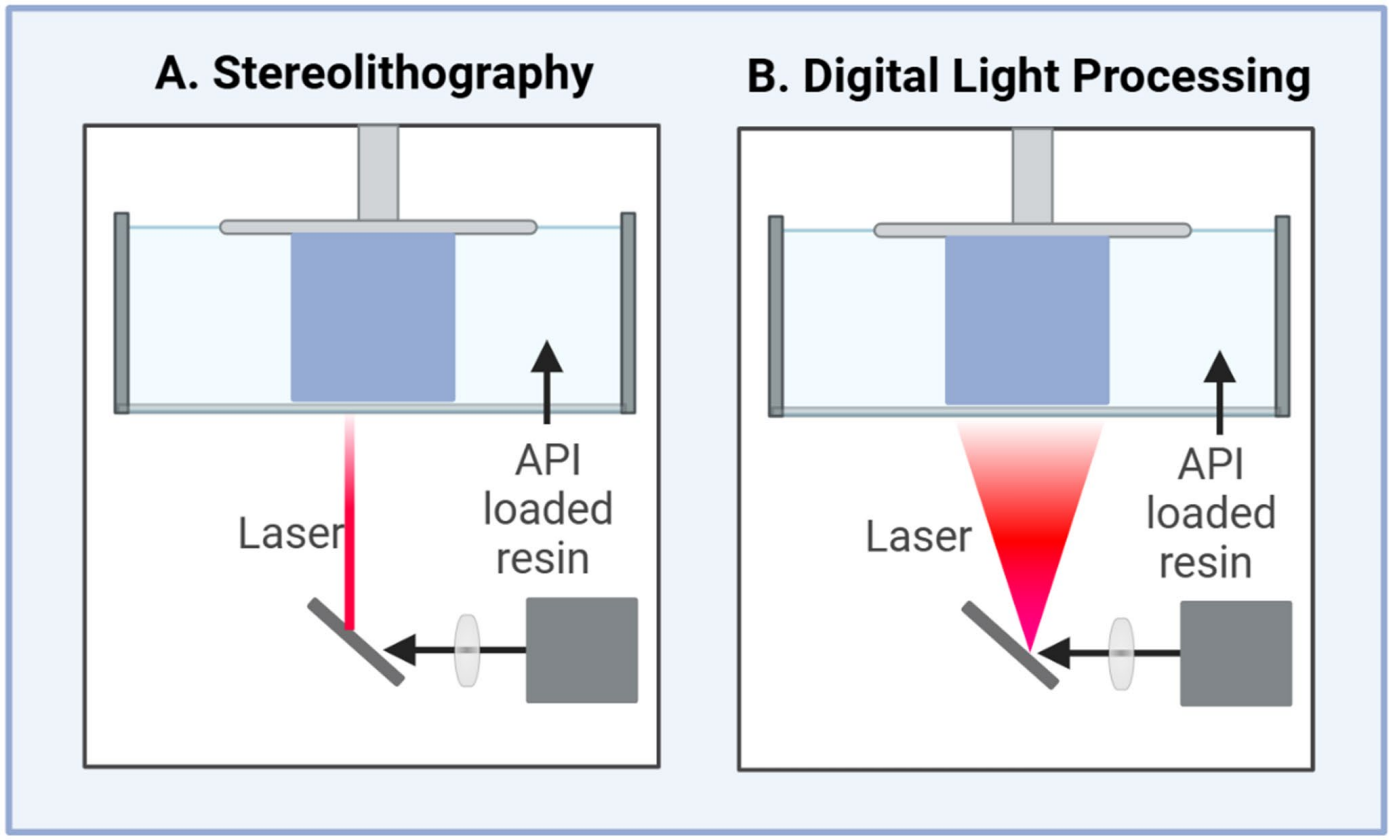
Simplified diagram for two types of vat photopolymerization-based 3D printing technology: Stereolithography (**A**) and Digital Light Processing (**B**)

SLA is a laser-based 3D printing technology that enables the fabrication of a pharmaceutical product via curing the photoreactive polymer layer by layer with the assistance of a single UV-laser beam (Fig. 4A). Historically, the SLA was the first invented 3D printing technology, introduced by Charles Hull in late 20th century, that became available for commercial use [119]. Due to its high resolution (30–100 μm), the SLA has been explored for the fabrication of scaffolds, personalized medical [117, 120] and drug-eluting devices in addition to pharmaceutical products [115]. Examples include polypill combinations of six drugs, controlled-release hydrogels and microneedles [42, 121, 122].

APIs are frequently blended into the resin and cured with the laser to make the desired final products. This resin should be characterized with specific CMAs to enable the photopolymerization process in the presence of excipients and/or APIs. The rheological properties of the liquid resin are identified as the most CMA that can modulate the CQAs of a pharmaceutical product (Table 5). Other attributes include but are not limited to polymer molecular weight, concentration and functional group type [123–125]. As an example, 3D-printed tablets using SLA with polyethylene glycol diacrylate (PEGDA), a photocurable monomer, exhibited an extended release profile of drugs, paracetamol and aminosalicylic acid, when a higher concentration of PEGDA was used (∼ 40% and 80% of aminosalicylic acid was released after 5 h with PEGDA 90% and 35%, respectively). This can be explained by the ability of drug molecules to diffuse easily outside of the matrix due to the poor crosslinking density of the tablets printed with low PEGDA concentration [121]. Furthermore, the photoinitiator concentration is another contributing factor to the crosslinking process, and it should be mixed at a certain ratio with the photocurable polymer [126].

The photopolymerization step of the SLA process varies based on both the composition of the resin and the 3D printing process parameters. Among the key CPPs for the SLA are laser power and exposure time. The laser beam triggers the photoinitiator to induce crosslinking between the reactive functional groups and controls the layer solidification mechanism as it moves across the resin in a single line. Many reports have reported the influence of laser power on dimensional accuracy and the layer thickness of the 3D-printed product [127]. In addition, there has been number of evidences showing a correlation between the laser power and the mechanical properties of the printed object [128]. These mechanical properties can become important when designing oral tablets with certain hardness and friability measures.

In a highly reactive environment, there are several possible scenarios for API to either degrade during the laser exposure or chemically react with the photocurable polymer. Xu et al. has reported the presence of Michael-Type Addition between amlodipine, an antihypertensive drug, and the functional groups of the PEGDA [118]. This type of reaction may cause a loss of drug efficacy and/or the formation of impurities, which can negatively compromise the product quality and safety.

##### Digital light processing

DLP can be considered an advanced version of the SLA. It, however, utilizes a specific light pattern reflected from digital micromirror devices (DMDs) into a photocurable resin [129]. This enables the curing of an entire layer when the UV light is emitted, introducing an efficient vat photopolymerization-based 3D printing technology (Fig. 4B). The DLP has been explored to fabricate contact lenses, tissue scaffolds and microneedles, along with a recent interest in developing oral pharmaceutical products [125, 130–132].

SLA and DLP construct 3D pharmaceutical products by a similar concept: the photopolymerization of a liquid resin, and thus, there are many parameters that overlap between them (Table 5). The CMAs of photocurable polymers discussed above will predominantly define the CPPs of the 3D-printing process, which centers around the light source intensity and exposure time [133, 134]. Nevertheless, there are a number of new studies that describe the influence of material parameters on the CQAs of pharmaceutical products fabricated with DLP-based 3D printing technology [135]. Kadry and his team published a report with significant insight on the effect of the photopolymer concentration, UV intensity, exposure time on the DLP and their implications on the printability of oral tablet loaded with theophylline [125]. In order to print tablets with defined shapes, the authors have reported the use of high polymer concertation and high UV intensity as well as longer exposure time. When the photopolymer (20%, w/v) was exposed to UV intensity of 12 mW/cm^2^ and exposure time of 35 s/layer, the fabricated tablets conformed with the weight variation and drug content according to the USP specifications < 905>. This underscores the reliability of the DLP-based 3D printing in products that can comply with the quality standards.

The balance between the printability of the photocurable polymer and the APIs should be accomplished to enable the 3D printing of pharmaceutical products with a therapeutic dose [135]. APIs with low potency, which are usually formulated in large quantities, might not be suitable for vat photopolymerization-based 3D printing technology due to the possible interference of drug molecules with the photopolymerization process and/or inability to fabricate products with satisfactory mechanical properties [136]. For instance, in the theophylline study above-mentioned, the authors stated that the drug properties (e.g., solubility, stability and loading efficiency) can become a barrier for 3D printing of pharmaceutical products with DLP. Due to the ongoing advancements in 3D printing technology and polymer chemistry, vat photopolymerization-based 3D printing is likely to enable the production of pharmaceutical products with a better safety profile, high biocompatibility and overcome some of the existing challenges with drug loading.

## Future outlook

New innovations in pharmaceutical products manufacturing have been encouraged by many regulatory bodies globally. In contemporary research, the 3D-printing technology has enabled pharmaceutical product development to take a step forward in personalized medicine, rare genetic disorder treatments and early-phase clinical trials development [137–139]. In pharmaceutical manufacturing, Triastek and Laxxon Medical are currently using 3D-printing technology for the advancement of 6 and 9 drug candidates, respectively, targeting a wide spectrum of diseases [140, 141]. 3D-printed pharmaceutical products have displayed bioequivalence to generic products and conform with pharmacopial specifications, as shown recently in the OPERA clinical trial [137, 142]. While successful attempts have been made, the regulatory framework for the 3D-printed pharmaceutical products is still unavailable. It is true that initiatives from regulatory authorities, such as the ETP program in the FDA, have established the recognition of the 3D-printed technology but there is a lack of detailed guidance to support the widespread implantation of 3D-printing technology in both industrial and PoC settings. Whether the 3D-printed pharmaceutical products should be accepted through a specific designated regulatory pathway or not is still largely unknown. What is well-known is that there is an urgent demand for a harmonized regulatory framework for pharmaceuticals manufactured with the 3D-printing technology [7, 15, 31, 143, 144].

To facilitate the regulatory adoption of 3D-printing in pharmaceuticals, a modular regulatory approach has been proposed. Instead of drafting one comprehensive guidance for the entire 3D-printing process, from the feedstock materials to the 3D-facribration process and quality control, the regulatory requirement can be divided into various components, each with its own set of regulatory specifications. Further, it is possible to manufacture feedstock materials by raw material suppliers under cGMP conditions and prepare them as pharmaceutical-grade feedstock material. These feedstock materials, referred to as ink cartridges, should adhere to strict quality specifications and comply with CMAs tailored for 3D-printing technology. A recent study has confirmed the feasibility of this setting [104]. Nevertheless, the regulatory framework should direct its focus to the 3D-printing process and establish guidance to identify the high-risk control points and CPPs for all the 3D-printing technologies, whether they are used at PoC or manufacturing facility. Emphasis should be dedicated to the underrated aspects of the 3D-printing process, such as the validation of 3D-printing process, the contaminations from the carryover feedstock materials and the post-processing conditions of the finished products [15, 23].

Despite the unique input of the 3D-printing technology in the product design and performance, all the 3D-printed pharmaceutical products should comply with existing quality standards of pharmaceutical dosage forms such as identification, assay and impurities. Nevertheless, its essential to expand quality specifications to address specific attributes of 3D-printed products. Examples of these attributes include porosity, layers adhesion, structure fidelity and mechanical properties [21–23, 143]. This scenario might be more relevant to large-scale manufacturing, where product quality is adequately controlled, but it may not be feasible for personalized medicine due to the variability in patient-specific needs. To overcome this hurdle, a case-by-case critical evaluation of the product can be considered and governed by a group of expert pharmacists at the PoC before the medicine is dispensed [37, 143]. The evaluation process has become even more feasible with the recent attempts of utilizing PAT to provide real-time evidence on the quality attributes of the 3D-printed pharmaceutical products. For instance, it was shown that in-line equipping of the FDM-based 3D printers with a near-infrared (NIR) spectrophotometer was able to predict the drug content in a batch of oral tablets [53]. Many other quality control measures are currently being investigated with the 3D printing technology and further details can be found in reference [143]. In addition, integrating PAT with 3D printers serves as a vital control strategy in large-scale manufacturing ensuring continuous production within the desired specifications and enabling quality to be built into every stage of the process, from the feedstock materials to the finished product [145]. Complementing this, the integration of artificial intelligence (AI) into 3D-printing processes for pharmaceutical manufacturing can facilitate the forecasting and optimization of formulation parameters, provide real-time process regulation and assist in the design of customized pharmaceutical products, thereby augmenting the overall efficiency of manufacturing process [146].

## Conclusion

The widespread adoption and advancement of 3D-printing technology in the pharmaceuticals is largely dependent on the establishment of a harmonized regulatory framework for licensing the 3DPPs. In the absence of comprehensive regulatory guidance, there will be a significant reluctance from pharmaceutical manufacturers and clinicians to fully unlock the potential of this technology. This review outlines the key quality attributes associated with feedstock materials and 3D-printing processes, supported by relevant examples that highlight their impact on the performance of 3D-printed products incorporated with APIs. These insights underscore the quality control measures that should be taken into consideration by regulatory authorities when evaluating 3DPPs. The development of a harmonized regulatory framework, along with clearly defined expectations from regulatory authorities, is imperative to ensure that 3DPPs meet the highest standards of quality, safety and efficacy. Looking forward, the successful implementation of such guidance will support the future of the 3D-printing technology in both pharmaceuticals and healthcare.

## Data Availability

During the preparation of this work, the author(s) used Open AI chatGPT in order to rephrase and restructure texts. After using Open AI chatGPT, the author(s) reviewed and edited the content as needed and take(s) full responsibility for the content of publication. The data that support the findings of this study are available from the corresponding author upon reasonable request.
