# Correction: Toward a harmonized regulatory framework for 3D-printed pharmaceutical products: the role of critical feedstock materials and process parameters

**DOI:** 10.1007/s13346-025-02000-w

**Published:** 2025-10-20

**Authors:** Riyad Alzhrani, Rawan A. Fitaihi, Majed A. Majrashi, Yu Zhang, Mohammed Maniruzzaman

**Affiliations:** 1 Department of Pharmaceutics, College of Pharmacy, King Saud University, Riyadh, 11451 Saudi Arabia; 2 Bioengineering Institute, King Abdulaziz City for Science and Technology (KACST), Riyadh, Saudi Arabia; 3 Pharmaceutical Engineering and 3D Printing (PharmE3D) Lab, Department of Pharmaceutics and Drug Delivery, School of Pharmacy, The University of Mississippi, University, MS 38677 USA

**Drug Delivery and Translational Research**

10.1007/s13346-025-01966-x

In the original online version of this article Rawan A. Fitaihi’s family name was spelled incorrectly. The original article was corrected.

